# Combining multi-scale modelling methods to decipher molecular motions of a branching sucrase from glycoside-hydrolase family 70

**DOI:** 10.1371/journal.pone.0201323

**Published:** 2018-08-01

**Authors:** Akli Ben Imeddourene, Jérémy Esque, Isabelle André

**Affiliations:** Laboratoire d’Ingénierie des Systèmes Biologiques et Procédés, LISBP, Université de Toulouse, CNRS, INRA, INSA, Toulouse, France; Universidade Nova de Lisboa Instituto de Tecnologia Quimica e Biologica, PORTUGAL

## Abstract

Among α-transglucosylases from Glycoside-Hydrolase family 70, the ΔN_123_-GB-CD2 enzyme derived from the bifunctional DSR-E from *L*. *citreum* NRRL B-1299 is particularly interesting as it was the first described engineered Branching Sucrase, not able to elongate glucan polymers from sucrose substrate. The previously reported overall structural organization of this multi-domain enzyme is an intricate U-shape fold conserved among GH70 enzymes which showed a certain conformational variability of the so-called domain V, assumed to play a role in the control of product structures, in available X-ray structures. Understanding the role of functional dynamics on enzyme reaction and substrate recognition is of utmost interest although it remains a challenge for biophysical methods. By combining long molecular dynamics simulation (1μs) and multiple analyses (NMA, PCA, Morelet Continuous Wavelet Transform and Cross Correlations Dynamics), we investigated here the dynamics of ΔN_123_-GB-CD2 alone and in interaction with sucrose substrate. Overall, our results provide the detailed picture at atomic level of the hierarchy of motions occurring along different timescales and how they are correlated, in agreement with experimental structural data. In particular, detailed analysis of the different structural domains revealed cooperative dynamic behaviors such as twisting, bending and wobbling through anti- and correlated motions, and also two structural hinge regions, of which one was unreported. Several highly flexible loops surrounding the catalytic pocket were also highlighted, suggesting a potential role in the acceptor promiscuity of ΔN123-GBD-CD2. Normal modes and essential dynamics underlined an interesting two-fold dynamic of the catalytic domain A, pivoting about an axis splitting the catalytic gorge in two parts. The comparison of the conformational free energy landscapes using principal component analysis of the enzyme in absence or in presence of sucrose, also revealed a more harmonic basin when sucrose is bound with a shift population of the bending mode, consistent with the substrate binding event.

## Introduction

Sucrose-utilizing α-transglucosylases from Glycoside-Hydrolase family 70 (GH70) [1], are extracellular bacterial enzymes of high molecular weight, typically in the range of 120–300 kDa, that naturally synthesize from sucrose substrate a wide variety of linear and branched α-glucan polymers differing in terms of type of glucosidic linkages, degree and spatial arrangement of branches, molecular size and physico-chemical properties [2–4]. For instance, GTF180-ΔN synthesizes a dextran polymer composed mainly of α-1,6 glucosidic linkages, GTF-A produces reuteran, an α-1,4 linked glucan [5], and GTF-SI forms mutan, an α-1,3 glucopolymer [3]. Another type of glucansucrases, the so-called dextransucrases (DSR), catalyze the formation of dextrans composed of a linear chain mainly formed of α-1,6 linked glucans on which are branched glucosyl chains through various types of α-1,2, α-1,3, and α-1,4 linkages [4]. One of these enzymes, DSR-E from *L*. *citreum* NRRL B-1299 [6], has been extensively studied in our group, revealing very original catalytic properties as it is able to synthesize dextran with high amounts of rare and non-digestible α-1,2 branching linkages. Interestingly, sequence and structure analysis combined to biochemical characterization revealed that this enzyme is bifunctional as it is composed of two catalytic domains, namely CD1 and CD2, linked by a glucan-binding domain (GBD) [6]. The catalytic domain CD1, in combination with the GBD at C-terminal extremity, is responsible for the α-1,6 glucan formation while the catalytic domain 2, combined to its GBD in N-terminal, is exclusively in charge of α-1,2 glucosyl branching onto dextran [7]. Based on its unique properties, GBD-CD2 was reported as being the first engineered Branching Sucrase (BRS), not able to elongate glucan polymers [6,8]. These results paved then the way for the discovery through data mining of novel natural GH70 branching sucrases specialized in dextran branching via α-1,2 or α-1,3 osidic linkages [9–11]. Products of these branching sucrases have proven to be of economical relevance as the presence of rare α-1,2 linkage leads to a resistance of mammalian digestive enzymes for the branched product and thus favors the growth of beneficial bacteria of the gut microbiome [12–16]. The α-1,3 branched glucan also shows interesting properties, especially in the inhibition of bacteria such as *Salmonella* or *Escherichia coli* [17].

**Fig 1:**
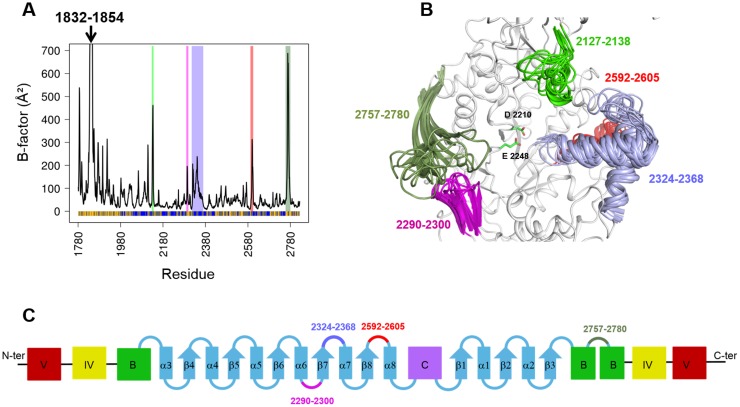
B-factors and flexible structural motifs along MD simulation. Panel (A) represents the average fluctuations of Cα atoms represented by B-factors per residue calculated along MD simulation of ΔN_123_-GBD-CD2. The strips in the background of the B-factor lines highlight structural motifs surrounding the active site, represented using the same color code on the panel (B), which represents the superimposed views of ten frames taken along the MD simulation. The highlighted regions are loop 2127–2138, helix-loop-helix motif corresponding to region 2324–2368 and its adjacent loop 2592–2605, then the 2290–2300 and 2757–2780 β-hairpin motifs in green, light blue, red, magenta and forest green, respectively. The shown side chains represent the two catalytic residues: the nucleophile D2210 and the acid base E2248. The panel (C) represents a schematic view of the five domains of ΔN123-GBD-CD2; detailed (β/α)_8_ barrel domain A with cyan color (helices represented as rectangles and β-sheets as arrows), the domains B, C, IV and V in green, magenta, yellow and red, respectively. The structural motifs surrounding the active site are represented with the same color code than panel (B).

**Fig 2:**
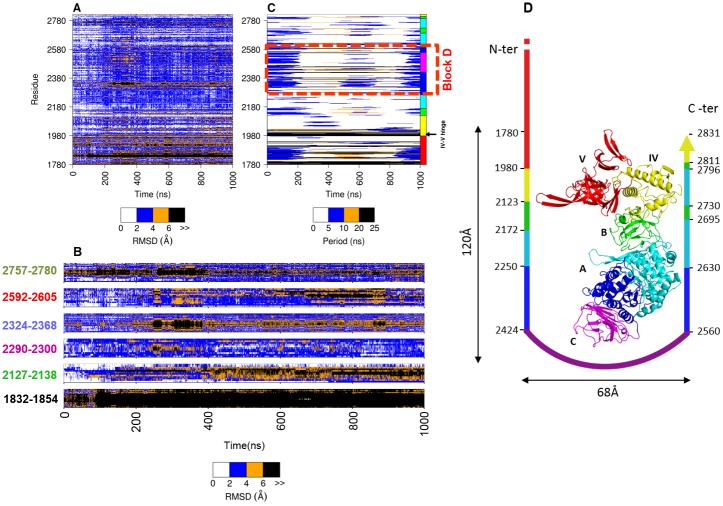
RMSD per residue and wavelet analysis. Residual RMSD (A) and wavelet (C) analysis for each amino acid residue as function of MD simulation time. The bottom legends show the color used to discriminate discrete RMSD values in Angström (panel A) or wavelet period in nanoseconds (panel B). The right-edge strip indicates the different structural domains that compose ΔN_123_-GDB-CD2 using the same color code as in panel D, the hinge between domains IV-V and the block D are highlighted on the right side. The bottom plot (B) shows an enlarged view of residual RMSD of flexible motifs surrounding the active site: loop 2127–2138, helix-loop-helix motif corresponding to residues 2324–2368 and its adjacent loop (residues 2592–2605), then the β-hairpin motifs composed respectively of residues 2290–2300 and 2757–2780, and the β-hairpin 1832–1854. The loop identifiers are colored in green, light blue, red, magenta, forest green and black respectively. The panel (D) highlights the five structural domains of ΔN_123_-GDB-CD2 with different colors: domains B, C, IV and V in green, magenta, yellow and red, respectively, the domain A is represented with two colors; blue for the block D and cyan for the rest of the domain. A schematic representation of ΔN_123_-GDB-CD2 structure is shown with delimitation of the different domains forming the U-shape.

The structural common characteristics of clan GH-H, that gathers enzymes from GH13, GH70 and GH77 families, is the (β/α)_8_ catalytic barrel domain, which is bound to N- and/or C-terminal domains. The type and number of other domains differ for each type of GH-H enzyme, suggesting that additional domains determine the reaction specificity of GH-H enzymes [18]. Nonetheless, the reaction specificity is largely determined by the catalytic domain. Moreover, N and C-terminal domains have many carbohydrate binding pockets that provide the enzymes with carbohydrate binding functionality [19–21]. Among the three-dimensional structures of sucrose-active transglucosylases from GH70 family available to date (GTF180-ΔN from *L*. *reuteri* (pdb entry: 3klk [22]); GTF-SI from *S*. *mutans* (pdb entry: 3aie; [23]); GTFA-ΔN reuteransucrase from *L*. *reuteri* (pdb entry: 4amc; [24]; DRS-M from *L*. *citreum* (pdb entry: 5lfc; [20]), that of the truncated variant ΔN123 GBD-CD2 (pdb entry: 3ttq; [7], 4ttu [25]) is the only one of a branching sucrase. In spite of their distinct reaction specificity, glucansucrases and branching sucrases share a common architectural organization composed of five domains, named A, B, C, IV and V, that altogether form an unusual global U shaped fold [7] (Figs 1 and 2). The first domain is the so-called domain A, which is composed of a (β/α)_8_ barrel that harbours the catalytic site. Domain B is located at the top of domain A and it consists of five or six antiparallel β-strands. Domain C, located at the bottom of the catalytic domain, forms eight antiparallel β-strands with a Greek key motif. Out of the five domains, it is the only one formed by a contiguous polypeptide stretch whereas all four remaining domains are built up of two discontinuous segments of the N- and C-terminal polypeptide chain. Domain IV is positioned between domains B and V, respectively. Its function is still obscure, except providing a linker between the catalytic core and the domain V [22]. Finally, domain V, also called Glucan Binding domain (GBD), is built up of an N-terminal segment alone in the case of ΔN123-GBD-CD2 or of both N- and C- terminal segments in other GH70 family members. Deletion of domain V has been shown to yield a fully active enzyme but altering the size of synthesized polymers in the case of DRS-S [26], GTF-180-ΔN [27], and DRS-M [20]. Conversely, truncation of domain V in GTF-A reuteransucrase from *Lactobacillus reuteri* 121 did not affect significantly size of polymer products [5]. However, the removal of ΔN123-GBD-CD2 glucan binding domain leads to an unstable enzyme displaying a very low activity toward sucrose [8]. Although the mechanism through which domain V assists catalysis is still poorly understood, it plays evidently a significant role in the control of oligosaccharide product length. Ito *et al* proposed that domain IV could serve as a structural hinge swinging the domain V toward and away from the catalytic domain [23]. This hypothesis was supported by the structure of GTF-180 solved in two distinct conformations [28]. Additionally, Small Angle X-ray scattering experiments suggested a bending of domain V in solution for DSR-M and GTF-180-ΔN to adopt a “horseshoe” shape [20,28]. Although evolutionary pathway that led to the structural organization of GH70 glucansucrases remains unclear, and in particular regarding the insertion of the enzyme precursor into domain V, it has been reported that domain V in ΔN123-GBD-CD2 shows sequence and structural similarity with the choline-binding domain of *Streptoccocus pneumoniae* LytA [29]; 29% identity; rmsd of 2.1 Å for 106 Cα atoms [22].

Although very appealing on a biotechnological point of view, detailed investigation and description of the structural organization and the functional role of macromolecular motions of these multi-domain enzymes are still missing to provide a comprehensive understanding of the enzyme reaction. Investigation at atomic level and in real time of macromolecular motions remains challenging for biophysical methods, although real breakthroughs have been achieved in the last years using a combination of different techniques (NMR, FRET, …) [30–34]. To overcome the lack of experimental information, molecular modelling techniques, such as molecular dynamics (MD) simulations, Normal Mode Analysis (NMA) and Essential Dynamics Analysis (EDA), remain the best way to access detailed dynamic information on macromolecular systems, in particular when large molecular systems and long timescale protein motions are considered [35,36].

Along this line, we report here for the first time the computational investigation of one member of GH70 family, the branching sucrase ΔN123-GBD-CD2, based on a combination of large scale MD simulations at the microsecond time range, NMA and EDA. This work provides a detailed picture at atomic level of the hierarchy of motions occurring along different timescales and how they are correlated in order to better understand their potential role on the reaction mechanism and substrate recognition of branching sucrases.

## Results and discussion

### Convergence of large-scale molecular dynamics simulation on ΔN123-GBD-CD2

To study dynamics of ΔN_123_-GBD-CD2, we performed an MD simulation of 1μs using the updated protein and glycan force fields. The whole system consisted of the protein, explicit representation of the solvent, and counterions. To collect statistics and ensure reliability of observed behaviours, MD simulations are usually run several times using different starting velocity distributions. However, in the present case, the large size of the ΔN_123_-GBD-CD2 simulated **s**ystem (136 311 atoms for the whole system) and the simulation time length (μs range) required to observe large-scale motions prevented us from repeating the simulations. To circumvent these limitations, convergence evaluation by different types of methods is generally required to ensure that an equilibrium state has been reached. Among these methods, Principal Component Analysis (PCA) is commonly used to evaluate the convergence of the conformational coordinates from MD simulations of proteins [37] or DNA [38], from replica exchange molecular dynamics [39], and from accelerated molecular dynamics [40]. The overlap of the principal component histograms is usually used to test the convergence of the MD simulation. This method is generally efficient to evaluate convergence of short simulations. For long MD simulations, the Kullback-Leibler Divergence (KLD), which is a measure of how two probability distributions overlap, performs usually better [40]. Therefore, considering the μs scale of the MD simulation on ΔN_123_-GBD-CD2, the KLD appeared more appropriate to quantify the convergence as a function of the simulation time using the Principal Component histograms corresponding respectively to the first or the second half of the trajectories (1 to 500ns vs 500ns to 1μs). The use of the two halves of the MD simulation was driven by the considerable computing needs required to carry out additional MD simulations at μs scale and supported by previous reports on various biological systems that compared the two halves of an MD simulation to evaluate the convergence of the simulation [37,41]. S1 Fig shows that KLD for the 3 first PC projections accounting for ~58% of the total motion reaches the equilibrium and remains around 0.03, indicating the reproducibility of the conformational space exploration on the two halves of 1μs MD simulation, and ensuring a reliable sampling.

As a check of the global dynamics evolution of the enzyme structure during the course of the MD simulation, the time evolution of the heavy atom RMSD resulting from alignment of the MD frames with respect to the X-ray structure (PDB code: 3TTQ) was calculated after least square fit (S2 Fig). The profile corresponding to the whole enzyme (S2 Fig, black line) indicates that during the first 100 ns of the MD trajectory, the RMSD fluctuates moderately (~2.5Å) from the X-ray structure. For the rest of the simulation, it increases up to ~ 4Å, reaching a spike to ~5Å during a few ns. These results indicate that the enzyme structure has undergone significant conformational changes during the course of the simulation. A detailed analysis of the simulation at molecular level showed a particularly high mobility of domain V, which is clearly illustrated by the decrease of RMSD values when excluding domain V in the calculation (S2 Fig, red line). In that case, the RMSD never exceeds 2.3Å from the X-ray reference structure and it remains constant ~ 1.8Å, suggesting that no important conformational change occurs. These results clearly demonstrate that domain V is by far the most flexible region in ΔN_123_-GBD-CD2, what was already experimentally suggested by SAXS experiments carried out with homologous GTF-180-ΔN enzyme for which domain V was seen pivoting between elongated and compact conformations [28].

### Local dynamics and loop flexibility of ΔN123-GBD-CD2 during the course of MD simulation

With the aim of investigating in details the local flexibility of the different domains of the enzyme, the B-factors were calculated along the MD trajectory on the basis of the Root Mean Square Fluctuations (RMSF) (Fig 1A). Overall, the B-factors calculated on enzyme Cα atoms from simulation frames were found to be in good agreement with the B-factors derived from X-ray structure (Pearson correlation coefficient of 0.71). Analysis showed that globally, domain C is the most stable domain while domain V is by far the most flexible region. As expected, the highest mobility is observed for the N-ter region 1832–1854, corresponding to the β-hairpin motif of domain V and for which the central turn region was unresolved in ΔN_123_-GBD-CD2 X-ray structures crystallized as a unique molecule in the asymmetric unit (S1 Table; pdb entries: 3TTQ [7] and, 4TTU, 4TTV and 4TVC [25]). Only in the case of an asymmetric unit containing four molecules, where each domain V is constrained by the C-ter domain of the neighbouring molecule, this region was structurally resolved (S1 Table; pdb entry:3TTO [7]). The domain IV presents moderate continuous fluctuations except for the loop 2105–2114. The B-factor values of amino acid residues from the (β/α)_8_ catalytic barrel (domain A) are variable with an alternation of rigid and flexible regions. Four flexible structural motifs surrounding the catalytic pocket and belonging to the domain A are identified in the B-factor plot (Fig 1A): the 2290–2300 and 2757–2780 β-hairpin motifs, that interact with each other via their central loops, the 2324–2368 helix-loop-helix motif, also called subdomain H1-H2 [7], and its adjacent loop 2592–2605 (Fig 1B). The B-factor plot revealed another highly flexible loop corresponding to residues 2127–2138 located at the top of the active site, and belonging to the N-terminal part of domain B (Fig 1B). Interestingly, the general relationship between the flexibility of the residues surrounding the active sites and promiscuity of enzymes, notably of ancestral enzymes, was reported in recent years [42–45]. In the case of ΔN123-GBD-CD2, one could assume that flexibility of the loops surrounding the active site could thus be related to the tremendous promiscuity observed for the enzyme toward the acceptor substrate. Indeed, in addition to the glucosylation of dextran chains, its natural acceptor substrate, ΔN123-GBD-CD2 enzyme has been shown to be able to glucosylate a variety of exogenous molecules such as a lightly protected disaccharide [46] or flavonoids [47]. Furthermore, the identified flexible loops surrounding the active site are believed to be non-essential for the catalytic machinery or the protein folding, and could thus be mutagenesis targets of interest to enlarge the repertoire of tolerated acceptor substrates without perturbing enzyme catalysis.

While RMSF (or B-factors) provide crucial information regarding the flexibility and the atomic fluctuations averaged along the MD simulation, notably to characterize movements of structural domains, they fail to provide insight on the time evolution of motions and the nature of the movement, such as Brownian or essential motions. Therefore, other analyses are required to capture the sequence and order of events occurring during MD simulation and to probe the dynamics amplitude and periodicity of flexible structural motifs of ΔN_123_-GBD-CD2, especially loops surrounding the catalytic gorge. The evolution of residual RMSD for each amino acid residue along simulation time is shown in Fig 2A. It reveals that residue motions mostly fluctuate with amplitudes between 2 to 4Å (blue color) from their average position along the MD simulation, with the exception of domain V that is found highly flexible along the simulation, notably the 1832–1854 β-hairpin motif, with many bands on the graph corresponding to the highest RMS deviation (> 6Å, colored in black in Fig 2B). The flexible structural elements located nearby the catalytic pocket identified by atomic fluctuation analysis in Fig 1 exhibit variable dynamic rates along the trajectory (Fig 2B). The RMSD of loop 2127–2138 belonging the domain B appears stable for the first 50ns of the trajectory, then oscillates essentially between 2 to 6Å throughout the rest of the simulation time. Likewise, the helix-loop-helix motif comprising residues 2324–2368 and the loop 2592–2605 appear stable at the beginning of the simulation until ~250ns, with a moderate deviation (0-4Å), pursued by an increase of the RMSD value particularly in region 2324–2368 for the next 150ns. The RMSD of the β-hairpin motif composed of residues 2290–2300 remains around 3Å during all the simulation, while the neighbouring β-hairpin motif formed by residues 2757–2780 is highly flexible with the same amplitude ~6Å over the entire trajectory. Interestingly, for these regions, the central residues (black bands in the middle) (Fig 2B) generally show the highest RMSD values, indicating that they are more flexible than the extremities.

To identify physically significant events occurring in MD simulations, not always easy to detect in the RMSD plot per-residue, we performed a Morlet wavelet analysis [48,49] that enables to highlight with higher sensitivity the periodic oscillations observed along the MD simulation. Besides specifying the temporal location of significant events, wavelet maps provide the timescale of those events. Large-scale motions are associated with low-frequency motions, while small scale motions are associated high-frequency motions. The examination of the Morlet oscillation map of ΔN_123_-GBD-CD2 (Fig 2C) revealed that events occurring between 0 and 10ns periods (in white and blue colors) dominate the plot. Interestingly, the region corresponding to the bottom part of the catalytic pocket, located between residues 2250 and 2630; so-called block D in Fig 2C (Block D: Down active site region comprising the 2250–2424 and 2560–2630 stretches of domain A and the entire domain C), behaves as one global wavelet spectra shape, i.e. events of 5-10ns period occurring during the first 200ns, then again in the middle of the simulation (between 500 and 700ns), and finally, during the last 100ns. Wavelet shape of domain B follows a similar behaviour.

Interestingly, the wavelet spectra of the upper part of the catalytic pocket in domain A (coloured in cyan in Fig 2B and 2D) is dominated by a fast dynamics period (in white color in Fig 2B) and thus differs significantly from the wavelet of the bottom part (in blue color in Fig 2B), suggesting a singular twofold behaviour of the catalytic domain A. Despite differences observed in the residual RMSD of domains IV and V, the wavelet map reveals a certain relationship between the dynamics of the two domains with continuous waves of slow events (periods between 20 and 25ns, black bands) on many residues from both domains. Noteworthy, the Wavelet analysis clearly exposes the hinge region between domains IV and V (Fig 2B) suggested earlier by Dijkstra and coworkers from X-ray structures of the GTF-180 solved in different conformations [28].

Morelet wavelet analysis appears here very efficient to detect important events in MD simulations that cannot be captured by traditional RMSD, or atomic fluctuations, and further provides periodic motion correlations between the different protein domains. However, Morelet wavelet analysis is unable to give the direction of the domain motions and thus their correlation, what is essential for understanding the conformational changes in proteins.

### Cross correlation of motions within ΔN123-GBD-CD2

**Fig 3:**
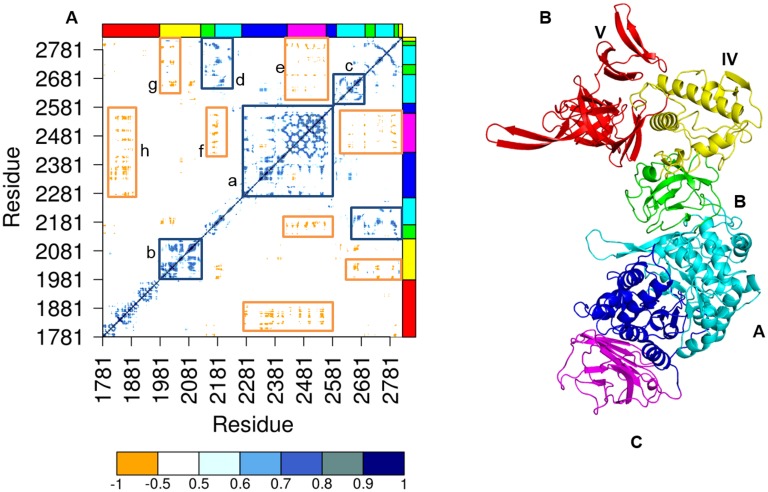
Dynamical cross correlation (DCC) analysis. (A) DCC map of ΔN_123_GBD-CD2 calculated from 1μs MD simulation. The color scale from orange to blue corresponds to discrete correlation coefficient values (DCC) from -1.0 to +1.0. The different structural domains of ΔN_123_GBD-CD2 are highlighted by strips on top and left side and using the same color code as in panel (B). Regions marked by the rectangles a-g are discussed in the main text. The panel (B) highlights the five structural domains of ΔN_123_-GDB-CD2 with different colors: domains B, C, IV and V in green, magenta, yellow and red respectively, the domain A is represented with two colors; navy blue for the block D and cyan for the rest of the domain A.

Correlated motions examine the relative motions of pairs of heavy atoms (or structural domains) during a simulation and identify pairs moving either in a correlated or anti-correlated fashion, what provides information regarding parts of the protein moving in tandem. The quantification of motion correlations between heavy atoms moving under multi-modal distributions provides the information on the motion direction vector, lacking in the Morelet analysis. In this approach, we first built a covariance matrix of atomic coordinates sampled from a MD trajectory, followed by the calculation of mean correlation coefficients. The cross correlation map of motion vectors calculated for heavy atom of ΔN_123_-GBD-CD2 along the 1μs MD simulation is shown in Fig 3A. In addition, the map also reveals the motion direction of the different structural domains along the MD trajectory. Analysis of the map indicates that domain C is the most internally correlated. The domain C also appears highly correlated to the bottom region of the active site from domain A, which together form the block D, corroborating the wavelet spectra of this region (Fig 3A, rectangle a). The block D is highly anti-correlated with the domain V (Fig 3A, rectangle g), what indicates an opposite motion direction of these two domains during MD simulation, and suggesting the existence of a global motion of domain V toward and away from the active site, in agreement with prior reports on GTF-180 [28]. Similarly to domain C, domains IV (rectangle b in Fig 3A) and the upper catalytic site of domain A (rectangles c and d in Fig 3A) undergo stronger intra-correlated motions than the ones observed for the domain V. Like domain V, the upper part of the catalytic site (rectangles e and f in Fig 3A) moves in the opposite way to the block D, reinforcing the existence of twofold dynamical direction of the domain A.

### Normal mode analysis and essential dynamics reveal new insights on global domain motions of ΔN123-GBD-CD2

Our goal here was to determine the global rigid body movements of the various domains from ΔN123-GBD-CD2. Normal Mode Analysis (NMA) and Essential Dynamics Analysis (EDA) are the major computational methods used to study the large-scale motions in biological molecules. The calculated modes (NMA or EDA) provide information on all the possible ways that a macromolecule can move, without however indicating how the molecular structure really moves in a given environment. It is thus not possible to determine which modes are functionally relevant from a set of calculated ones [50], requiring therefore experimental data to sort out representative movements [51,52]. The analysis of the eigenvalue dispersion of NMA and EDA shown in S3 Fig reveals the existence of two regimes of eigenvalue distribution: the three first modes constitute the slowest motions and the other modes correspond to the intermediate dynamics. The same discrimination between the contribution of the different modes to the global motion was done by Bahar and coworkers [53]. Remarkably, the two methods (NMA and EDA) present the same intercept of eigenvalue distribution, proving that the amplitude of the motions driven by normal modes and essential dynamics are nearly similar in our study.

**Fig 4:**
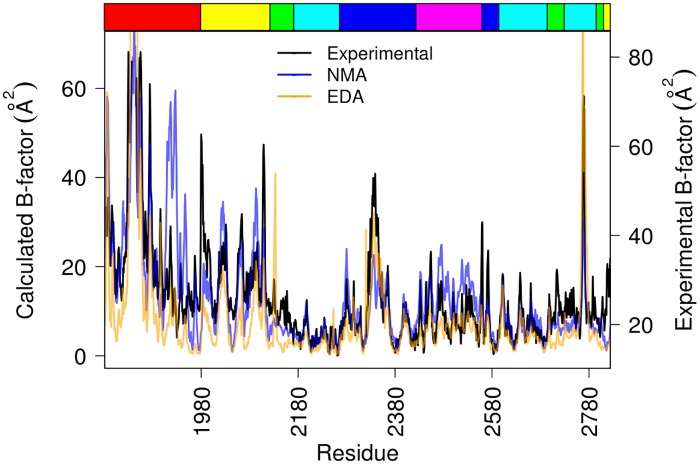
Comparison between computed and experimental B-factors. B-factors were calculated from the three first modes of NMA (blue), and EDA (orange) and plotted against experimental B-factors derived from X-ray structures of ΔN_123_-GBD-CD2 (black) as a function of amino acid residues. The top edge strip shows the different domains of ΔN_123_-GBD-CD2 using the same color code as in Figs 2 and 3.

To ensure the experimental relevance of NMA and EDA sampling, a comparative analysis of B-factor values per residue was carried out between these computational methods and eight crystallographic structures of the ΔN_123_-GBD-CD2 available in the Protein DataBank (S1 Table). Interestingly, the calculated atomic fluctuations of Cα for the three first modes of NMA or EDA (more than 50% of total motions for both), when considered together, have a good correlation with respect to the crystallographic B-factors, i.e. R-Pearson coefficients of 0.68 and 0.76 for NMA and EDA, respectively (Fig 4). Moreover, including remaining modes did not alter the general shape of the B-factor profile, but decreased the correlation coefficient.

Compared to experimental values, calculated atomic fluctuations from NMA are relatively poorly reproduced for domains IV and V with an R-Pearson coefficient of 0.53, while the correlation coefficient remains unchanged (R = 0.78) for EDA. The poor reproducibility of experimental data by normal modes comparatively to essential modes could be due to the method bases. Indeed, motions resulting from NMA represent only the harmonic dynamics in vacuum and thus do not include the solvent surrounding the protein (water molecules and ions), whereas EDA derived from MD simulation takes into account periodic boundary and solvent effect [54], capturing the anharmonicity of the protein dynamics. From NMA results, the most divergent region is the edge β-hairpin of the solenoid fold from domain V, situated between residues 1930 and 1956, probably due to the fact that the direction of the motion may vary along the conformational change (anharmonicity), what is poorly reproduced by global harmonic normal mode dynamics [52]. The comparison of crystallographic B-factors and those derived from EDA or NMA confirms the good sampling of the conformational space by the three first modes, which cover 58% and 71% of eigenvalues from EDA and NMA, respectively, attesting of the robustness of the methods to compute rigid body motions.

**Fig 5:**
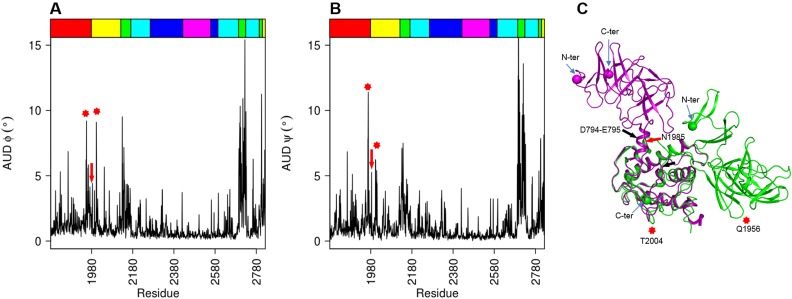
Average unsigned dihedral backbone angles. The panels (A, B) illustrate the average unsigned (absolute) deviation (AUD) of Φ, Ψangles, respectively, resulting from the three first modes of NMA. The top edge strip shows the structural domains of ΔN_123_-GBD-CD2 using the same color code as in Figs 2 and 3. The red stars indicate the hinge regions Q1956 and T2004 from N-ter to C-ter respectively, the red arrow show the N1985 hinge residue. The panel (C) represents the superimposition of IV and V domains of the GTF180-ΔN (pdb entry: 3KLK) in magenta and ΔN123-GBD-CD2 (pdb entry: 3TTQ) in green. The spheres show the N-ter and C-ter residues of each enzyme. The location of hinge regions discussed in this paper are indicated by the red arrow and stars. The black arrow indicates the hinge region (D794-E795) identified in GTF180-ΔN [18,28].

More detailed information on the internal flexibility of the structural domains may be acquired by examining the variations in the backbone dihedral angles resulting from the low frequency normal modes [55–57]. The calculation of the ψ and Φ backbone angles absolute deviation from the average structure of the three first modes shown in the Fig 5 enables to clearly pinpoint the pivots of rotating domains in ΔN_123_-GBD-CD2. Domain B displays the most variable torsional backbone angles, what supports the assumed role as hinge of this domain. The pivot region located between domains IV and V is situated between residues Q1956 and T2004. Inspection of the hinge residues reveals that Q1956 acts as the hinge of the edge β-hairpin of the solenoid fold from domain V described above, while the T2004 ensures a local dynamics of the loop connecting IV and V domains (Fig 5C). Remarkably, a hinge residue (N1985) comparable to that found in GTF-180 (residues D794-E795) [18,28] is identified in ΔN_123_-GBD-CD2. Interestingly, the most stable backbone torsion angles are seen in the block D, what suggests a quasi-rigid behaviour [58] of the block, and confirms the earlier results derived from MD simulation analysis.

**Fig 6:**
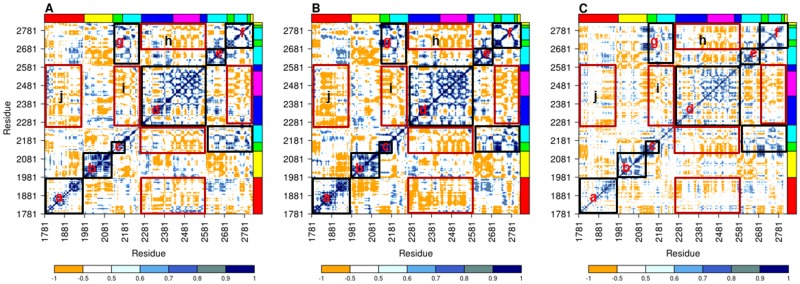
Dynamical cross correlation from theoretical methods against experimental data. DCC map of ΔN_123_-GBD-CD2 calculated from EDA (A), NMA (B), and derived from X-ray (C). The color scale from orange to blue corresponds to discrete correlation coefficient values (DCC) from -1.0 to +1.0. The structural domains of ΔN_123_-GBD-CD2 are shown using the same color code as in Fig 3. Parts marked by the rectangles a-j are discussed in the main text.

To explore further the nature of the global movements taking place within the enzyme structure, we performed an inter-residue correlation analysis from X-ray structures or from normal mode dynamics. This latter was widely used to analyze in more details the correlated motions occurring between residues within macromolecules [53,54,56,59,60]. Worth pointing, dynamical inter-residue correlation heat maps represented in Fig 6 show a remarkable agreement between the cross correlation motions calculated from the three first modes of EDA and NMA, or derived from X-ray structures (Table 1), providing a high level of confidence in the dynamics of structural domains coming out of these analyses. Furthermore, the heat maps clearly outline the various hinge regions of collective motions experienced by the enzyme, which are confirmed by the average unsigned deviation of backbone Ψ and Φ torsions angle derived from the MD average structure of ΔN_123_-GBD-CD2.

**Table 1:** Pearson correlation coefficient (PCC) between DCC maps. PCC values were calculated between pairs of DCC maps shown in Fig 6.

	NMA	EDA	X-ray
NMA	1	0.87	0.75
EDA	--	1	0.70
X-ray	--	--	1

For all analyses (EDA, NMA and X-ray), all domains display a positive intra-domain correlation motion along the diagonal (a, b, c, d, e, and f in Fig 6) indicating that residues that belong to a same domain, and that are continuous in the protein sequence, move in general along the same direction. Some domains present stronger intra-domain correlated motions (beyond the diagonal) such as IV and C (b and upper part of rectangle d in Fig 6), and are conserved according to three methods. Conversely, domain A presents some differences of motion correlation depending on the method, mostly in the rectangle d where more anti-correlated motions are viewed for EDA and X-ray analysis. Moreover, domain A shows a net split into two regions, corresponding respectively to the bottom part of the catalytic gorge highly correlated to domain C, and which form together the block D (rectangle d in Fig 6), and the upper part of the active site (rectangles g, e and f in Fig 6). Indeed these two regions display anti-correlated motions with each other (rectangles i and h in Fig 6) exposing here again the twofold behaviour of the catalytic domain A detected in earlier analyses. In contrast to intra-domain global dynamics, the cross correlation heat map reveals that some domains are completely independent of each other (negatively signed cross correlations) such as the block D versus the domain V (rectangle j in Fig 6). Detailed analysis of the heat map shows a fine-tuned partitioning of the dynamics of ΔN_123_-GBD-CD2 that goes beyond the organization of the enzyme in five structural domains based on secondary and tertiary structural motifs defined earlier by X-ray crystallography studies [7].

**Fig 7:**
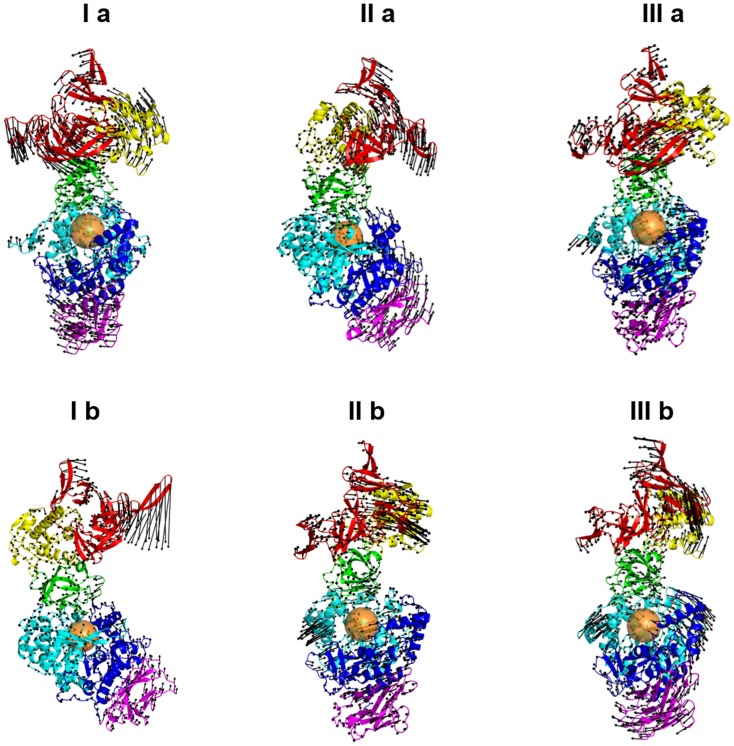
Large scale collective motions from normal or essential dynamics modes. View of the three first normal modes of ΔN_123_-GBD-CD2, corresponding respectively to the twist (I a), bend (II a) and wobble (III a) modes and three first essential dynamics modes corresponding respectively to the bend (I b), wobble (II b) and twist (III b). The black arrows point to the motion direction for each mode and the length of the arrows illustrates the amplitude of the movements. The orange spheres indicate the location of the catalytic gorge.

We then examined at three-dimensional level the significance of these correlated motions. The three universal low frequency normal modes or essential dynamics corresponding to large scale collective motions detected for ΔN_123_-GBD-CD2 are shown in Fig 7. The observed movements are associated to twisting, bending and wobbling modes [36,50,61]. The twisting mode is mainly characterized by a counter clockwise rotation of the domains A and C, and in opposite direction of domains IV and V; the bending mode corresponds to the movement of domains IV and V with respect to the domains A and C; the wobbling mode involves nearly all the protein with the exception of domain B. Remarkably the order of the occurrence of three first modes differs depending on the method (NMA or EDA). The order of three lowest modes is relayed to the initial coordinates used for NMA calculation [60] or EDA [62]. Taken together, the three lowest modes provides a comprehensive picture of the collective motions experienced by ΔN_123_-GBD-CD2. Its behaviour is similar to that of proteins composed of two globular domains linked by a flexible hinge [50]. In the case of ΔN_123_-GBD-CD2, the flexible hinge linker is the domain B, which connects two globular blocks: the first block composed of domains A and C, the second block comprised of domains IV and V (Fig 7). Whereas the hinge region linking domains IV and V (residue N1985) was experimentally proposed in homologous GTF180 enzyme [28], the function of domain B as a hinge cleft was yet unreported to our knowledge, and inaccessible via conventional simulations.

### Effect of sucrose binding on the local dynamics of ΔN_123_-GBD-CD2

**Fig 8:**
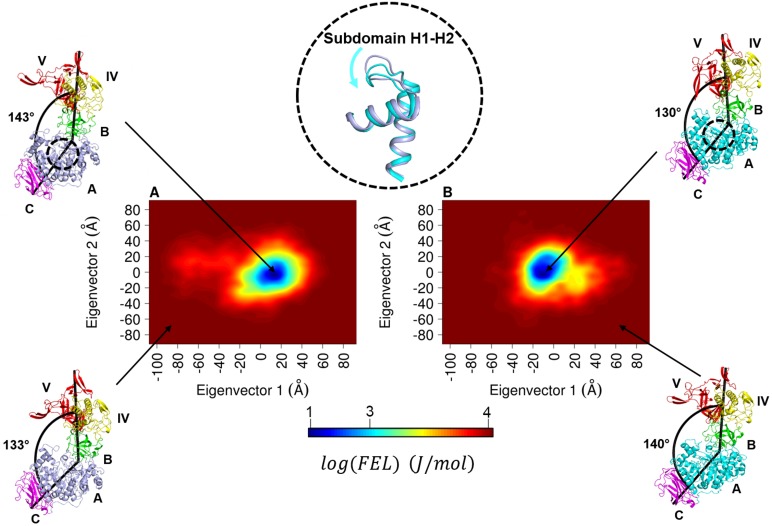
Free energy landscape (FEL) of ΔN_123_-GBD-CD2. FEL in free form (A) and in complex with sucrose (B) were determined using as reaction coordinates the projection of the first and second principal components. The bottom legend shows the color scale of the logarithm of FEL in J. mol^-1^. Structural snapshots taken from the low or high energy regions pointed by the arrows are shown. The top center structural motif represents the subdomain H1-H2 structures from energetic wells of free-ligand (light blue) or complex (cyan) simulations. An empirical angle formed between the Cα atoms of N7 (domain V), the E912 (domain A) and N738 (domain C) was defined for each conformation to illustrate the bending mode.

To explore the potential effect of sucrose binding on the conformational space exploration, dynamics, and flexibility of structural motifs surrounding the active site of ΔN_123_-GBD-CD2, we performed an MD simulation of 100ns of the enzyme in complex with sucrose, the donor substrate. In the absence of an experimental structure of ΔN123-GBD-CD2 in complex with sucrose, only MD simulations enable to investigate conformational rearrangements occurring upon sucrose binding and predict contribution of amino acid residues from the catalytic pocket to the productive recognition of sucrose. The enzyme:sucrose complex was obtained by superimposing ΔN123 GBD-CD2 with GTF-180, the high conservation of residues interacting with the sucrose from GTF-180 [22] in ΔN123-GBD-CD2 ensures the high confidence of the sucrose docking. The monitoring of the RMSD (S4 Fig) of the enzyme and the ligand along the 100ns trajectory provides information regarding the stability of the complex and the proper binding of sucrose in productive conformation. Interestingly, like for the free enzyme MD simulation, the RMSD plot shows that the domain V remains the most flexible region within the complex system. In order to compare behaviour of ΔN_123_-GBD-CD2 in free form and in complex with sucrose, reduction of the number of frames and their dependence on the read-out time step and the length of simulation is required [63]. Free energy landscape based on the kernel density estimation of the two first principal components is often used to extract most relevant structures from MD simulation [63–67]. The free energy landscape maps calculated for the free enzyme and enzyme:sucrose complex reveal in both cases only one energetic basin (Fig 8). While the ordinate coordinate of the energetic basins, corresponding to second principal component (Wobbling mode) and the third PC corresponding to the twisting mode (S5 Fig), remains similar in the two simulations, the first eigenvector coordinates representing the binding mode in PCs shifts to negative values in the MD simulation of the enzyme: sucrose complex. The consequences of the basin displacement on the global motion of the enzyme is discussed in the next section.

**Fig 9:**
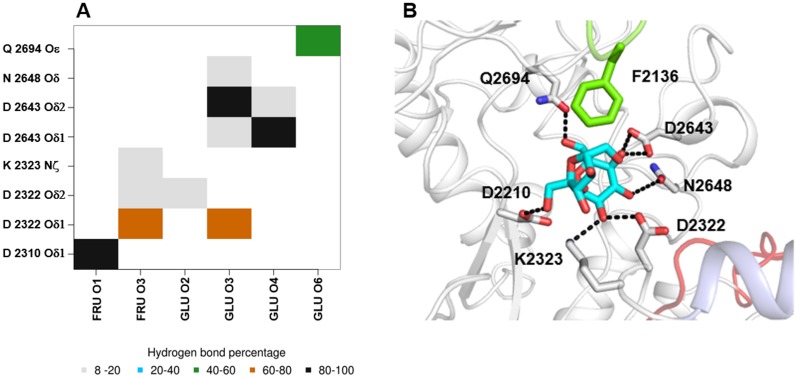
Main interactions between sucrose and amino acid residues in the active site. Map of hydrogen bonding interactions occurring between ΔN123-GBD-CD2 and sucrose in clusters taken from the energy landscape basins (ΔG < 100 J. mol^-1^) (A). The x and y axis indicate the atoms from sucrose and the enzyme involved in hydrogen bonding interactions. The color code indicates the hydrogen bond occurrence percentage over the cluster. In (B) panel are illustrated the important interactions observed between sucrose and amino acids in the catalytic pocket.

The comparison of the clusters taken from the energy landscape basins (ΔG < 100 J. mol^-1^) of the free and complex simulations shows that the calculated B-factors (S6 Fig) are highly similar, with a very good Pearson correlation coefficient of 0.95. The five highly flexible structural motifs surrounding the catalytic site, identified in the MD simulation of the free enzyme (Fig 3), have different behaviour in the presence of sucrose. Whereas, the 2290–2300 and 2757–2780 β-hairpins undergo same dynamics amplitude in both MD simulations, the three other motifs are less flexible. Interestingly, the 2127–2128 loop is fully stabilized along the simulation in the presence of sucrose, viewed by a significant decrease of the flexibility between the free form and the complexed one. This stabilization is mostly due to hydrophobic stacking interaction of F2136 side chain with the fructosyl moiety of the sucrose that is observed in all frames taken from the low energetic basin (Fig 9B). The subdomain H1-H2 also appears less flexible in the presence of sucrose bound in the active site, notably the 2324–2336 helix. The enhanced rigidity of this structural motif is due to the hydrogen bonding interaction between the glucosyl moiety from sucrose and residues D2321 and K2322 located upstream of the helix (Fig 9A and 9B). The behaviour of the H1-H2 subdomain reduces significantly the flexibility of the adjacent 2592–2605 loop in the simulation of the enzyme: sucrose complex, which is sandwiched between the H1-H2 domain and the 2634–2651 loop containing the D2643 and the N2648 residues that form two hydrogen bonds with the O3 and O4 of the glucosyl moiety from sucrose. Two other residues stabilize the sucrose ligand in the active site and help to maintain a productive conformation. The first one is the residue Q2394 that forms a hydrogen bond with the O6 of the glucosyl. The second one is the nucleophile residue D2210 which shows an interesting behaviour, i.e. one oxygen atom of the carboxyl group forms a hydrogen bond with the O1 of the fructose moiety, whereas the second one is oriented toward the C1 of the glucosyl, favouring thus the nucleophilic attack.

Comparison of the two X-ray structures of ΔN_123_-GBD-CD2 (unbound and bound enzyme: glycerol) revealed that subdomain H1-H2 (residues 2324–2368) displays the highest structural divergence (S7 Fig). Furthermore, the corresponding motif in DSR-M undergoes a similar shift when comparing free enzyme (pdb entry: 5LFC) and sucrose-inactive mutant complex (pdb entry: 5O8L) [20]. Similarly, the structure of GTF-180-ΔN in complex with sucrose (pdb entry: 3HZ3) shows a shift of this subdomain. In overall, analysis of the different available X-ray structures suggests that the shift of the H1-H2 subdomain in direction of the catalytic pocket is only observed when sucrose (3HZ3 and 5O8L) or glycerol (3TTQ, 3KLK) molecules is bound to the catalytic site. The examination of subdomain H1-H2 conformations shown in Fig 8, provides that this subdomain undergoes in MD simulations the same shift observed in X-ray structures when enzyme is bound to sucrose. These results strongly suggest the implication of the subdomain H1-H2 dynamics in the recognition of sucrose and its stabilisation in the active site.

#### Effect of sucrose binding on the global dynamics of ΔN_123_-GBD-CD2

As described in the previous section, the first principal component presents a shift in the kernel density center when comparing the free enzyme and the enzyme: sucrose complex simulations, moreover its distribution appears more harmonic in the complex system. Thus the essential dynamics extracted from the first PC appears the appropriate tool for understanding the structural meaning of the KDE shift and harmonicity. The cross correlation atomic motion maps (S8 Fig) obtained from essential dynamics indicate that the major difference between complex and free-ligand forms reside in the block D, with increased intra-domain correlated motions in the complex PCs. Remarkably, the Pearson correlation coefficient points out that the essential dynamics atomic cross correlation of the complex is more correlated to the normal mode of the free enzyme form (R = 0.95) than to the essential dynamics of the free enzyme system (R = 0.86). The similarity between the essential dynamics and normal mode analysis supports the harmonicity observed on the distribution of first eigenvector from the enzyme:sucrose system. The analysis of the enzyme structures extracted from the energetic basins from free and complex systems shows clearly that the thermodynamics equilibrium (conformer population) is shifted toward the closed form when the enzyme is bound to sucrose (Fig 8). In this closed conformation, the sucrose is then positioned near the nucleophile D2210 to enable catalysis (S9 Fig). To illustrate the observed bending mode, an empirical angle formed between the Cα atoms of N7 (domain V), the E912 (domain A) and N738 (domain C) was defined, and was found to be lower in the complexed form (Fig 8). Altogether these results suggest a role of the global dynamics and especially the twofold behaviour of domain A on sucrose recognition and its catalytic transformation by ΔN_123_-GBD-CD2.

## Conclusion

In summary, large-scale MD simulation of 1μs and normal mode analysis was carried out for the first time on a branching sucrase from GH70 family, namely the ΔN123-GBD-CD2. By combining multiple analyses and different metrics, we were able to uncover specific motions and events occurring at different timescales, dynamical correlations existing between the different amino acid residues and more generally, study how they impact the global dynamics of the enzyme and substrate recognition. Overall, our simulations show a fine-tuned partitioning of the dynamics that goes beyond the organization of the enzyme in five structural domains. Correlated motions, corroborating the conformational organization of the enzyme in crystallographic lattices, revealed a cooperativity of low frequency twisting, bending and wobbling modes leading to the movement of two blocks formed respectively by domains A and C and domains IV and V, and connected via the domain B which forms a flexible structural hinge reported here for the first time to our knowledge. The flexibility of the domain V is found to be by far the most flexible structural domain in the enzyme and an integral part of global dynamics, swinging toward and away from the catalytic site, around a clearly identified hinge region (residues 1956 and 2004) between domains IV and V. Interestingly, normal modes and essential dynamics underlined a two-fold dynamic of catalytic domain A, pivoting about an axis splitting the catalytic gorge in two parts. Furthermore, catalytic pocket is surrounded by highly flexible loops that could play a key role in the acceptor promiscuity of ΔN123-GBD-CD2. The probed local and global dynamics of the enzyme:sucrose complex confirm the implication of the loops surrounding the active site and the role of global binding mode in the stabilization of sucrose in the catalytic site and assisting its productive binding. Inferred information on long timescale motions occurring in ΔN123-GBD-CD2 would have been otherwise inaccessible via biophysical methods, notably the function of domain B, which long remained totally obscure. In perspective, the fundamental knowledge gathered in this study will be of central importance for the design of enzyme variants with improved properties for biotechnological applications.

## Material and methods

### 3D-model construction

The three-dimensional modelling of ΔN123-GBD-CD2 was based on the 1.8Å resolved crystallographic structure of ΔN123-GBD-CD2 (pdb entry: 3ttq [7]) for which the missing loop 1839–1849 was rebuilt using the FREAD server [68]. The enzyme:sucrose complex was constructed by extracting sucrose molecule from the X-ray structure of homologous GTF180 in complex with sucrose (pdb entry 3hz3 [22]). The H++ webserver [69] was used to determine the protonation state of ionisable residues at pH 5.4 at which ΔN123-GBD-CD2 exhibits the optimum of activity [70]. The heptacoordinated calcium and sodium ions located at the interface of domains A and B or domains V and IV, respectively, were considered in MD simulations.

### Molecular dynamics simulations

MD simulations were performed using the AMBER ff14SB force-field [71] for enzymes and GLYCAM_06j-1 [72] for sucrose ligand. The NAMD program [73] was used for simulations with the free enzyme whereas pmemd.CUDA [74] which supports the mixed scaling of 1–4 non-bonded electrostatic and van der Waals terms [75] in amino acids and sugars was used for simulations on enzyme:sucrose complex. MD simulations were carried out at constant temperature (303K) and pressure (1bar) using the Berendsen algorithm [76]. The integration time-step was 2fs and covalent bonds involving hydrogens were constrained using SHAKE [77]. The non-bonded pair-list was updated heuristically. Long-range electrostatic interactions were treated using the Particle Mesh Ewald (PME) approach [78]. Non-bonded interactions were treated with a 9Å direct space cut-off. All enzyme systems were neutralized with Na^+^ ions [79] (minimal salt condition), in explicit TIP3P water molecules [80]; the primary boxes were rectangular with solvent extending 10Å around the enzymes. The water molecules and counterions were energy-minimized and equilibrated at 100K around the constrained solute for 100ps in the NVT ensemble; the entire system was then heated incrementally over 100ps from 100 to 300K in 5K steps with harmonic positional restraints of 25.0kcal/mol/Å^2^ on the solute atoms. The MD simulations were continued in NPT, without notable change in volume. The positional restraints were gradually removed over 250ps and followed by the production phase. MD snapshots were saved every 10ps. The MD simulations were carried out for a total of 1μs for the free enzyme and 100ns for the sucrose bound complex.

#### Normal mode analysis

The initial enzyme coordinates were taken from the crystal structure of ΔN123-GBD-CD2 (pdb entry: 3ttq). Hydrogen atoms were added, and system topology was generated using the CHARMM-GUI web-based graphical interface [81]. The energy was minimized by the conjugate gradient method using the CHARMM36 program [82] with CHARMM36 force field [83]. Harmonic constraints were applied to heavy atoms to allow smooth minimization without abrupt deviation from the crystallographic structure for the first 1000 steps. The positional restraints were gradually decreased over 100 steps, the constant forces were 250, 100, 50, 25, 10 and 5 kcal/mol/Å^2^, followed by 20 000 steps of a non-constrained minimization. The DIMB method [84,85] was used to compute low-frequency normal modes in the Cartesian space (ϖ < 10 cm^−1^, 26 modes), with a convergence of 0.08 for the eigenvectors. The maximum size of diagonalized blocks was 1500·3 atoms, with 15952 atoms of ΔN123-GBD-CD2, the required memory space was 64.8 Gigabytes. The time consumption was 250 hours. The first six modes, which correspond to global translation and rotation of the whole enzyme, were removed.

### Trajectory analysis

#### Principal component analysis and MD convergence

Principal Component Analyses (PCA) were performed using CPPTRAJ [86]. The coordinate covariance matrix of only heavy atoms was calculated using an offset of 10 frames. Each snapshot of the trajectory was aligned to the overall average coordinates in order to remove global rotational and translational motions. The projection along these 20 first eigenvectors for each coordinate frame from the simulation trajectory was then calculated. First, the eigenmode calculation was done considering the overall MD simulation in order to study the global motion of the enzyme. Next, the overlap of PCA histograms corresponding to the first and second half of each MD simulation trajectory was used to probe the system convergence using the Kullback-Leibler Divergence (KLD) metric [87] as described by Cheatham’s group [38].

#### Root Mean Square Deviation and atomic fluctuations

The Root Mean Square Deviation (RMSD) of Cα atoms over the whole enzyme was calculated for each MD simulation. Furthermore, the average per-residue (residual) RMSD was computed in order to follow the deviation at the residue level during the course of the MD simulation. In order to remove translational and rotational motions, the MD snapshots were rms-fitted onto the X-ray structure. The Root Mean Square Fluctuation (RMSF) permits the measure of the atomic average mobility during MD simulation. In this work, the average mass-weighted fluctuations of Cα atoms and the B-factors were calculated for each residue using the following equations:

$$RMSF=\sqrt{\frac{1}{\text{nsteps}}\sum_{i=1}^{nsteps}{\left\|r_{i}\left(t\right)-\langle r_{i}\rangle \right\|}^{2}}$$

and

$$B-factor\;=\;{RMSF}^{2}\left(\frac{8}{3}\right)\pi^{2}$$

Where *r*_*i*_ is the position of atom (*i*) at time (*t*), and 〈*r*_*i*_〉 is the average position of the atom.

#### Dynamical Cross-Correlation Matrices

Analysis of normal modes, essential dynamics trajectories and X-ray structure was performed in terms of covariance matrix *c*_*ij*_ and Dynamical Cross-Correlation Matrices (DCCM) [88]. All coordinates were translated and rotated by means of a least-square-fitting procedure using heavy atoms of backbone and side chains to align the equilibrated starting conformation of simulated data or X-ray structure (pdb entry: 3TTQ). The covariance matrix *c*_*ij*_ for the position vectors of two residues *i* and *j* in the fitted structure is calculated as:

$$c_{ij}\;=\;\langle \left(r_{i}-\langle r_{i}\rangle )(r_{j}-\langle r_{j}\rangle \;\right)\rangle \;=\;\langle r_{i}\;r_{j}\rangle -\langle r_{i}\rangle \langle r_{j}\rangle$$

The cross-correlation matrix elements *C*_*ij*_ are defined by the following equation:

$$C_{ij}=\frac{c_{ij}}{\sqrt{c_{ii}}\;\sqrt{c_{jj}}}=\frac{\langle r_{i}r_{j}\rangle -\langle r_{i}\rangle \langle r_{j}\rangle }{\sqrt{[(\langle r_{i}^{2}\rangle -{\langle r_{i}\rangle }^{2})\left(\langle r_{j}^{2}\rangle -{\langle r_{j}\rangle }^{2})\right]}}$$

Cross-correlation coefficients range from a value of −1 (completely anti-correlated motions) to a value of +1 (completely correlated motions).

#### Wavelet analysis

In our study, we used the new implementation of wavelet analysis [49] in CPPTRAJ 2017 software to perform the wavelet analysis using Fast Fourier Transform (FFT) algorithm [89] on MD simulation trajectory. Translational and rotational motions were removed as described above. Wavelet analysis contains two main steps which perform Continuous Wavelet Transform (CWT) and statistical significance testing as described by Torrence and Compo [90]. For each heavy atom, Morelet CWT [91] is calculated over a given scale ranging from 0.2 to 25 ns and by multiplying with a correction value of 1.01. All wavelet analyses were done using χ^2^ = 1.6094 which is the minimum acceptable value for running a significance test at 99% confidence level.

#### Free-energy landscape

The free-energy landscape of a macromolecule can be obtained using a MD simulation sampling method that allows to explore the conformations near the native state structure [67]. Here, we constructed the free-energy landscape along the two first Principal Components (PCs) using the following equation:

$$G_{a}\;=\;-kTln\frac{P(q_{a})}{P_{max}(q)}$$

Where *k* is the Boltzmann constant, *T* is the temperature of the simulation (303Kelvin), *P*(*q*_*α*_) is an estimation of the probability of an α state and the *P*_*max*_(*q*) is the probability of the most probable state. Considering the two reaction coordinates (two first PCs), the free-energy landscape is obtained from the joint kernel density estimation of the distribution *P*(*q*_*i*_,*q*_*j*_) of the system using the fast computation of multivariate Kernel estimators [92].

## Supporting information

S1 Fig MD simulation convergence. Kullback-Leibler Divergence of Principal Component projection histograms was calculated from first (1 to 500ns) and second half (500ns to 1μs) of MD simulation trajectories vs time for the three first principal components (colored in black, yellow and orange, respectively).
(PDF)

S2 Fig Quantitative measurement of ΔN_123_-GBD-CD2 MD conformational motions during MD simulation of 1μs carried out in water. Heavy atom Root Mean Square Deviation of the whole enzyme (black line) and enzyme without domain V (red line) with respect to X-ray structure were calculated as function of simulation time.
(PDF)

S3 FigNormalized eigenvalues versus Mode number.Eigenvalues of each mode were normalized over the total motion (eigenvalue_(i)_ /$\;\sum_{n\;=\;i}^{i\;=\;1}\;$ eigenvalue) and plotted against Mode number of NMA (black dots) and EDA (red triangles), respectively.(PDF)

S4 Fig Quantitative measurement of ΔN_123_-GBD-CD2-sucrose complex MD conformational motions during MD simulation of 100ns carried out in water. Heavy atoms Root Mean Square Deviation of the whole enzyme (black line), enzyme without domain V (red line) and sucrose (green line) with respect to X-ray structure were calculated as function of simulation time.
(PDF)

S5 Fig Free energy landscape (FEL) of ΔN_123_-GBD-CD2. FEL in free form (A) and in complex with sucrose (B) were determined using as reaction coordinates the projection of the second and third principal components. The bottom legend shows the color scale of the logarithm of FEL in J. mol^-1^.
(PDF)

S6 Fig Average mass-weighted fluctuations of Cα atoms. B-factors were calculated as function of enzyme amino acid residues along 100ns MD simulation of ΔN_123_-GBD-CD2 in complex with sucrose (yellow line) or the free-ligand MD simulation (black line). The bottom strip represents the secondary structures of X-ray ΔN_123_-GBD-CD2 for reference: helix (blue), sheet (orange) and coil (grey). The strips in the background of the B-factors lines highlight structural motifs surrounding the active site, represented using the same color code as Fig 3B in the main text. The highlighted regions are loop 2127–2138, helix-loop-helix motif corresponding to region 2324–2368 and its adjacent loop 2592–2605, then the 2290–2300 and 2757–2780 β-hairpin motifs in green, light blue, red, magenta and forest green, respectively.
(PDF)

S7 Fig Superimposition of the two solved structures of ΔN_123_-GBD-CD2. The helix-loop-helix motifs corresponding to residues 2324–2368 are highlighted in light blue (pdb entry: 3ttq) and cyan (pdb entry: 4ttu). The side chains in sticks represent the catalytic residues: the nucleophile D2210 and the acid base E2248. The orange sphere is shown for reference to locate the active site.
(PDF)

S8 Fig Dynamical inter-residue correlation map of ΔN_123_-GBD-CD2. Maps were calculated from free ligand EDA (A), NMA (B), and from enzyme-sucrose complex EDA (C). The color scale from orange to blue corresponds to discrete correlation coefficient values (DCC) from -1.0 to +1.0. The structural domains of ΔN_123_-GBD-CD2 are shown using the same color code as in Fig 3B in the main text.
(PDF)

S9 Fig Conformation of sucrose in the active site. The closed form (A) and the open form (B) were extracted from essential dynamics analysis. The distances in Angstrom between the carboxyl oxygen atom of the nucleophile D2210 and C1 of sucrose are shown for each form.
(PDF)

S1 Table List of ΔN123-GBD-CD2 X-ray structures available in the Protein Data Bank. (PDF)
