# Supplementary material for: Combining multi-scale modelling methods to decipher molecular motions of a branching sucrase from glycoside-hydrolase family 70
Source: PLoS One. 2018 Aug 1;13(8):e0201323. doi: 10.1371/journal.pone.0201323 (PMC6070258; doi:10.1371/journal.pone.0201323)
Supplement: S1 Fig — Kullback-Leibler Divergence of Principal Component projection histograms was calculated from first (1 to 500ns) and second half (500ns to 1μs) of MD simulation trajectories vs time for the three first principal components (colored in black, yellow and orange, respectively). (PDF) [file pone.0201323.s001.pdf]

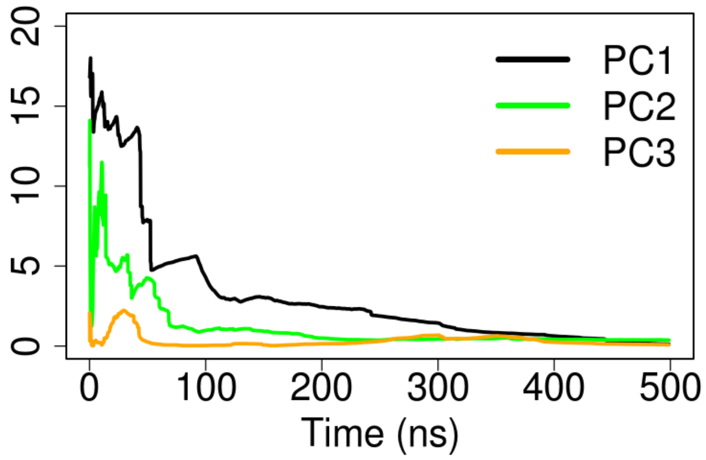

S1 Fig. MD simulation Convergence. Kullback-Leibler Divergence of Principal Component projection histograms was calculated from first (1 to 500ns) and second half (500ns to 1μs) of MD simulation trajectories vs time for the three first principal components (colored in black, yellow and orange, respectively).
