# Supplementary material for: Combining multi-scale modelling methods to decipher molecular motions of a branching sucrase from glycoside-hydrolase family 70
Source: PLoS One. 2018 Aug 1;13(8):e0201323. doi: 10.1371/journal.pone.0201323 (PMC6070258; doi:10.1371/journal.pone.0201323)
Supplement: S2 Fig — Heavy atom Root Mean Square Deviation of the whole enzyme (black line) and enzyme without domain V (red line) with respect to X-ray structure were calculated as function of simulation time. (PDF) [file pone.0201323.s002.pdf]

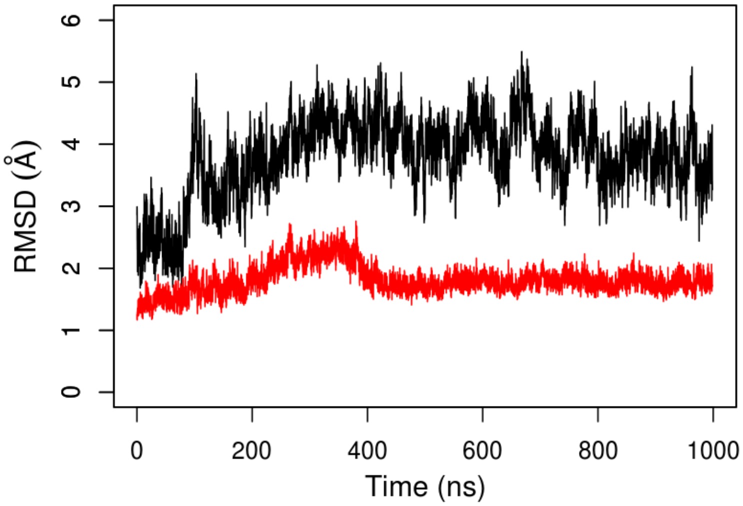

S2 Fig. Quantitative measurement of ΔN_{123}-GBD-CD2 MD conformational motions during MD simulation of 1μs carried out in water. Heavy atom Root Mean Square Deviation of the whole enzyme (black line) and enzyme without domain V (red line) with respect to X-ray structure were calculated as function of simulation time.
