# Supplementary material for: Combining multi-scale modelling methods to decipher molecular motions of a branching sucrase from glycoside-hydrolase family 70
Source: PLoS One. 2018 Aug 1;13(8):e0201323. doi: 10.1371/journal.pone.0201323 (PMC6070258; doi:10.1371/journal.pone.0201323)
Supplement: S3 Fig — Eigenvalues of each mode were normalized over the total motion (eigenvalue(i) /∑n=ii=1 eigenvalue) and plotted against Mode number of NMA (black dots) and EDA (red triangles), respectively. (PDF) [file pone.0201323.s003.pdf]

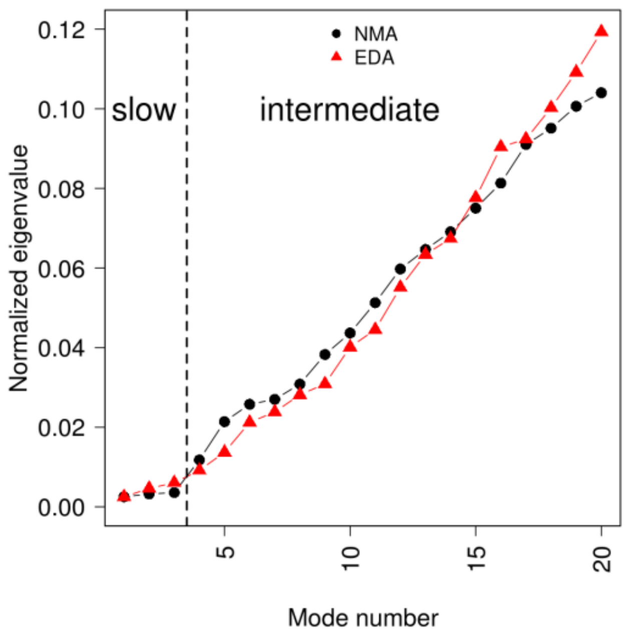

S3 Fig. Normalized eigenvalues versus Mode number. Eigenvalues of each mode were normalized over the total motion (eigenvalue $_{(i)}$ / $\sum_{n=i}^{i=1}$ eigenvalue) and plotted against Mode number of NMA (black dots) and EDA (red triangles), respectively.
