# Supplementary material for: Combining multi-scale modelling methods to decipher molecular motions of a branching sucrase from glycoside-hydrolase family 70
Source: PLoS One. 2018 Aug 1;13(8):e0201323. doi: 10.1371/journal.pone.0201323 (PMC6070258; doi:10.1371/journal.pone.0201323)
Supplement: S4 Fig — Heavy atoms Root Mean Square Deviation of the whole enzyme (black line), enzyme without domain V (red line) and sucrose (green line) with respect to X-ray structure were calculated as function of simulation time. (PDF) [file pone.0201323.s004.pdf]

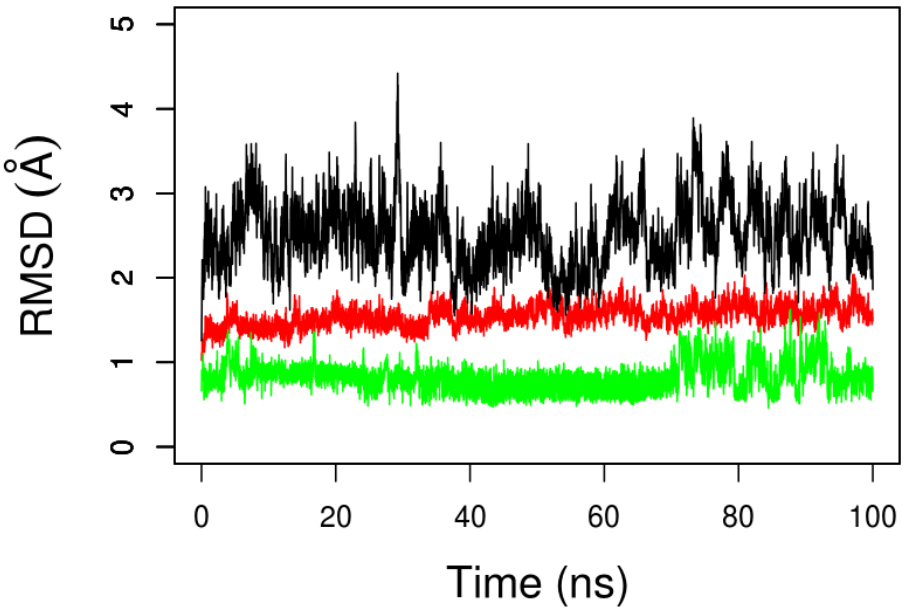

S4 Fig. Quantitative measurement of ΔN_{123}-GBD-CD2-sucrose complex MD conformational motions during MD simulation of 100ns carried out in water. Heavy atoms Root Mean Square Deviation of the whole enzyme (black line), enzyme without domain V (red line) and sucrose (green line) with respect to X-ray structure were calculated as function of simulation time.
