# Supplementary material for: Combining multi-scale modelling methods to decipher molecular motions of a branching sucrase from glycoside-hydrolase family 70
Source: PLoS One. 2018 Aug 1;13(8):e0201323. doi: 10.1371/journal.pone.0201323 (PMC6070258; doi:10.1371/journal.pone.0201323)
Supplement: S5 Fig — FEL in free form (A) and in complex with sucrose (B) were determined using as reaction coordinates the projection of the second and third principal components. The bottom legend shows the color scale of the logarithm of FEL in J. mol-1. (PDF) [file pone.0201323.s005.pdf]

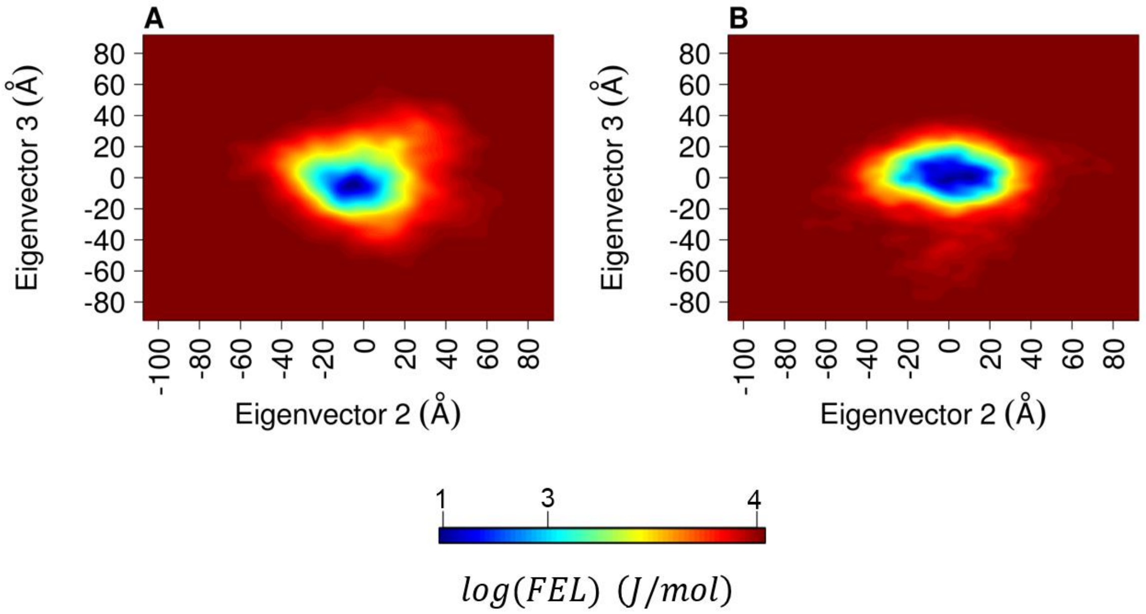

S5 Fig. Free energy landscape (FEL) of ΔN_{123}-GBD-CD2. FEL in free form (A) and in complex with sucrose (B) were determined using as reaction coordinates the projection of the second and third principal components. The bottom legend shows the color scale of the logarithm of FEL in J. mol^{-1}.
