# Supplementary material for: Combining multi-scale modelling methods to decipher molecular motions of a branching sucrase from glycoside-hydrolase family 70
Source: PLoS One. 2018 Aug 1;13(8):e0201323. doi: 10.1371/journal.pone.0201323 (PMC6070258; doi:10.1371/journal.pone.0201323)
Supplement: S6 Fig — B-factors were calculated as function of enzyme amino acid residues along 100ns MD simulation of ΔN123-GBD-CD2 in complex with sucrose (yellow line) or the free-ligand MD simulation (black line). The bottom strip represents the secondary structures of X-ray ΔN123-GBD-CD2 for reference: helix (blue), sheet (orange) and coil (grey). The strips in the background of the B-factors lines highlight structural motifs surrounding the active site, represented using the same color code as Fig 3B in the main text. The highlighted regions are loop 2127–2138, helix-loop-helix motif corresponding to region 2324–2368 and its adjacent loop 2592–2605, then the 2290–2300 and 2757–2780 β-hairpin motifs in green, light blue, red, magenta and forest green, respectively. (PDF) [file pone.0201323.s006.pdf]

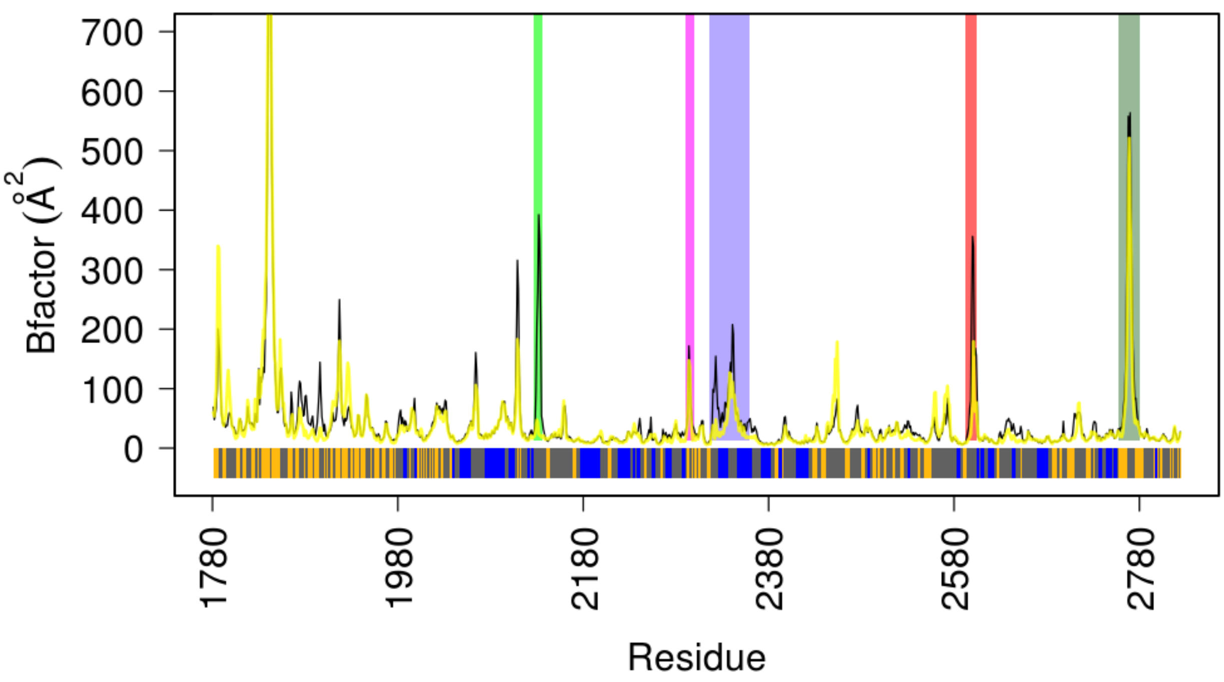

S6 Fig. Average mass-weighted fluctuations of Cα atoms. B-factors were calculated as function of enzyme amino acid residues along 100ns MD simulation of ΔN_{123}-GBD-CD2 in complex with sucrose (yellow line) or the free-ligand MD simulation (black line). The bottom strip represents the secondary structures of X-ray ΔN_{123}-GBD-CD2 for reference: helix (blue), sheet (orange) and coil (grey). The strips in the background of the B-factors lines highlight structural motifs surrounding the active site, represented using the same color codeas Fig 3B in the main text. The highlighted regions are loop 2127-2138, helix-loop-helix motif corresponding to region 2324-2368 and its adjacent loop 2592-2605, then the 2290-2300 and 2757-2780 β-hairpin motifs in green, light blue, red, magenta and forest green, respectively.
