# Supplementary material for: Combining multi-scale modelling methods to decipher molecular motions of a branching sucrase from glycoside-hydrolase family 70
Source: PLoS One. 2018 Aug 1;13(8):e0201323. doi: 10.1371/journal.pone.0201323 (PMC6070258; doi:10.1371/journal.pone.0201323)
Supplement: S7 Fig — The helix-loop-helix motifs corresponding to residues 2324–2368 are highlighted in light blue (pdb entry: 3ttq) and cyan (pdb entry: 4ttu). The side chains in sticks represent the catalytic residues: the nucleophile D2210 and the acid base E2248. The orange sphere is shown for reference to locate the active site. (PDF) [file pone.0201323.s007.pdf]

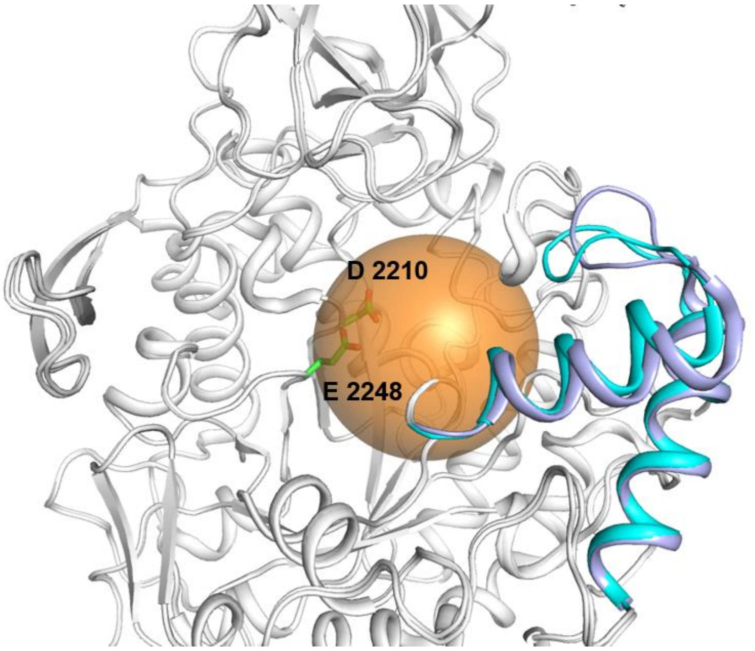

S7 Fig. Superimposition of the two solved structures of ΔN$_{123}$-GBD-CD2. The helix-loop-helix motifs corresponding to residues 2324-2368 are highlighted in light blue (pdb entry: 3ttq) and cyan (pdb entry: 4ttu). The side chains in sticks represent the catalytic residues: the nucleophile D2210 and the acid base E2248. The orange sphere is shown for reference to locate the active site.
