# Supplementary material for: Combining multi-scale modelling methods to decipher molecular motions of a branching sucrase from glycoside-hydrolase family 70
Source: PLoS One. 2018 Aug 1;13(8):e0201323. doi: 10.1371/journal.pone.0201323 (PMC6070258; doi:10.1371/journal.pone.0201323)
Supplement: S8 Fig — Maps were calculated from free ligand EDA (A), NMA (B), and from enzyme-sucrose complex EDA (C). The color scale from orange to blue corresponds to discrete correlation coefficient values (DCC) from -1.0 to +1.0. The structural domains of ΔN123-GBD-CD2 are shown using the same color code as in Fig 3B in the main text. (PDF) [file pone.0201323.s008.pdf]

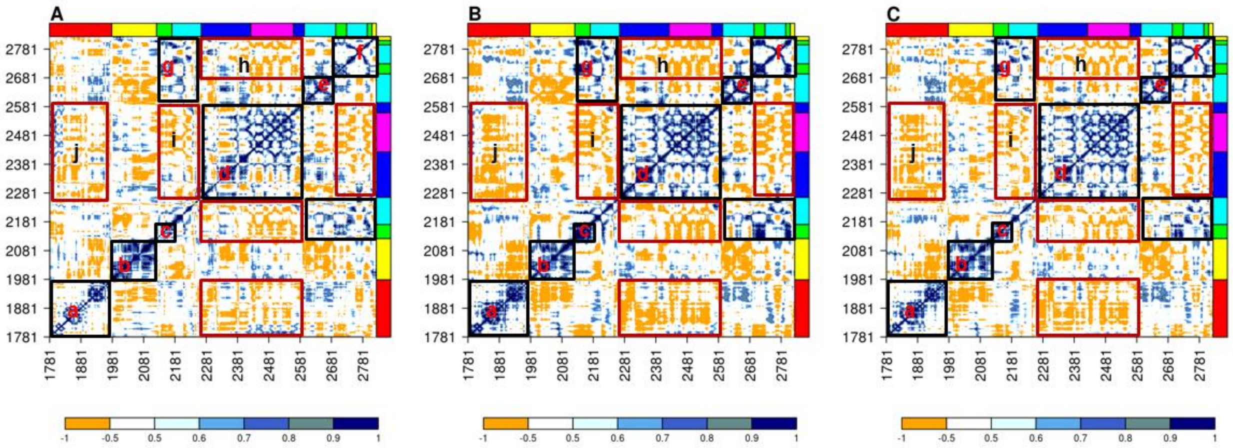

S8 Fig. Dynamical inter-residue correlation map of ΔN$_{123}$-GBD-CD2. Maps were calculated from free ligand EDA (A), NMA (B), and from enzyme-sucrose complex EDA (C). The color scale from orange to blue corresponds to discrete correlation coefficient values (DCC) from -1.0 to +1.0. The structural domains of ΔN$_{123}$-GBD-CD2 are shown using the same color code as in Fig 3B in the main text.
