# Supplementary material for: Combining multi-scale modelling methods to decipher molecular motions of a branching sucrase from glycoside-hydrolase family 70
Source: PLoS One. 2018 Aug 1;13(8):e0201323. doi: 10.1371/journal.pone.0201323 (PMC6070258; doi:10.1371/journal.pone.0201323)
Supplement: S9 Fig — The closed form (A) and the open form (B) were extracted from essential dynamics analysis. The distances in Angstrom between the carboxyl oxygen atom of the nucleophile D2210 and C1 of sucrose are shown for each form. (PDF) [file pone.0201323.s009.pdf]

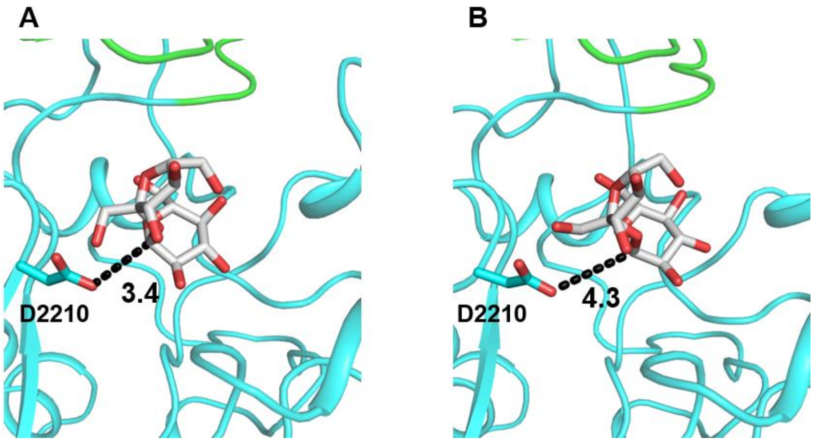

S9 Fig. Conformation of sucrose in the active site. The closed form (A) and the open form (B) were extracted from essential dynamics analysis. The distances in Angstrom between the carboxyl oxygen atom of the nucleophile D2210 and C1 of sucrose are shown for each form**.**
