# Supplementary material for: Combining multi-scale modelling methods to decipher molecular motions of a branching sucrase from glycoside-hydrolase family 70
Source: PLoS One. 2018 Aug 1;13(8):e0201323. doi: 10.1371/journal.pone.0201323 (PMC6070258; doi:10.1371/journal.pone.0201323)
Supplement: S1 Table — (PDF) [file pone.0201323.s010.pdf]

S1 Table. List of ΔN123-GBD-CD2 X-ray structures available in the Protein Data Bank.

PBD entry	Resolution (Å)	Number of molecules in the asymmetric unit	Co-crystalized molecules
3TTQ	1.9	1	Ca^{2+}, Na^{+}, Glycerol and tetramethylene glycol
3TTO	3.3	4	Ca^{2+} and Glycerol
4TTU	2.2	1	Ca^{2+}, Na^{+}, α-D-glucose and Di(hydroxyethyl)ether
4TVD	2.3	1	Ca^{2+}, Na^{+}, α-D-glucose, β-D-glucose<br>and Di(hydroxyethyl)ether
4TVC	1.9	1	Ca^{2+}, Na^{+}, α-D-glucose
